# 360° approach to the patient with mite allergy: from scientific evidence to clinical practice

**DOI:** 10.3389/falgy.2024.1298816

**Published:** 2024-02-06

**Authors:** Antonio Nieto-García, Eva Abel-Fernández, María Nieto-Cid, Fernando Pineda de la Losa

**Affiliations:** ^1^ Pediatric Allergy and Pneumology Unit, La Fe Hospital, Valencia, Spain; ^2^ La Fe Health Research Institute, Valencia, Spain; ^3^ Applied Science Department, Inmunotek SL, Alcalá de Henares, Spain; ^4^ Allergy Service, University Hospital of La Plana, Vila-real, Spain

**Keywords:** precision medicine, molecular diagnosis, allergen specific immunotherapy (ASIT), mites allergy, allergic rhinitis, asthma

## Abstract

In the recent years, several important advances have been made in the diagnosis of allergy using molecular techniques. The aetiological diagnosis of allergy using molecular components of allergens allows a more precise definition of the patient's IgE repertoire. Precision medicine is a structural model aimed at personalising healthcare and places the patient at the centre of the specialist's decision-making process. To this end, an accurate characterisation of the external exposome at a molecular level and their putative role as clinically relevant allergens is essential to elucidate the phenotypic diversity of atopic disease, with a view to personalising diagnosis and therapy. It has been proposed a decision algorithm, the Top-Down approach, where the clinical history is set first and is followed by the use of skin tests or specific IgE techniques, which facilitates the clinicians to make decisions. The therapeutic intervention driven by the standard diagnostic approach, but supported by these innovative tools, can lead to a better phenotyping of highly complex patients, and a more appropriate prescription of AIT. To this end, the allergen extracts used for diagnosis require to be of proven quality and contain the most relevant allergens. Likewise, allergen vaccines must gather efficacy, safety, duration, and patient compliance, hence the demand for new vaccines to overcome these drawbacks.

## Precision medicine

With the motto “Pathways from precision medicine to personalised healthcare in allergy and asthma”, the new goal of the European Academy of Allergy and Clinical Immunology is to offer individually tailored healthcare solutions to improve the life of the allergic patient.

The phrase “It's far more important to know what person has the disease than what disease the person has” is attributed to Hippocrates (460-370 bC) and would be the atavistic precursor of the concept of “personalized medicine”, which was explicitly formulated In 2012 Mirnezami R et al., published an article entitled “Preparing precision medicine” (1). They described how a 35-year-old Japanese patient diagnosed with non-small-cell lung cancer, was shown to respond to a specific agent, selected on a basis of a panel of genetic variants that could predict whether the disease would respond to treatment, and whose administration led to the remission of the cancer in less than a year, having a skin rash as the only side effect. This case illustrates the idea of personalised medicine, a combination of established clinic pathological indices linked to cutting-edge molecular profiles to create diagnostic, prognostic, and therapeutic strategies, precisely tailored to the needs of each patient.

Precision medicine can therefore be defined as a structural model aimed at personalising healthcare, with medical decisions or products tailored to a patient at a very detailed level (1). In this regard, the aetiological diagnosis of allergy using molecular components of allergens allows a more precise definition of the patient's IgE repertoire (2).

Allergy is a discipline of health where precision medicine has already proven its suitability. In the recent years, several important advances have been made in the area of molecular diagnosis in allergy (3–5), and the set of techniques available for allergists are currently much powerful than in the 2000's. Component-resolved diagnostics (CRDs) have expanded rapidly, and their high specificity fits well into the overall concept of precision medicine. In addition, “computer-aided diagnosis” has significantly modified the general behaviour of physicians (6) allowing to identify new possibilities derived from the complexity of IgE profile interpretation, especially in polysensitised patients with sensitisation to genuine allergenic or cross-reactive components (7).

The rapprochement between clinical practice and laboratory research has improved the diagnosis and treatment of allergic patients, where knowledge of patient's sensitization profile is derived from the combination of skin prick tests and specific IgE assays, driving to a highly personalised diagnosis and custom-made allergen immunotherapy (AIT).

## Diagnosis

The availability of highly specialised mono- and multi-parametric platforms helps allergists to have an advanced diagnostic arsenal. The therapeutic intervention driven by the standard diagnostic approach, but supported by these innovative tools, can lead to a better phenotyping of highly complex patients, and a more suitable prescription of AIT. The selection of appropriate allergen immunotherapy to treat the allergic disease becomes more complex when the results of the standard diagnostic tests show a broad polysensitisation profile in the patient, and the clinical history does not provide enough evidence to make a correct decision. This may occur in a relatively high proportion of patients, as polysensitisation is more common than monosensitisation.

AIT is grounded in the fundamental assumption that patients primarily exhibit sensitization to major allergens rather than minor allergens (8). Instead of adhering to the traditional administration of AIT to patients solely sensitized to the “major allergens”, a more progressive strategy involves the identification and incorporation of the “minor allergens” into commercially available AIT preparations (8).

The use of molecular diagnostic platforms, especially with multiplexed tools supposes some advantages over the use of allergen extracts, offering information about the status of sensitization, allowing the discrimination between genuine sensitization and cross-reactivity in polysensitized patients, helping to narrow the primary approach obtained from the standard diagnosis based on allergen sources and offers improved specificity and sensitivity of IgE test. Singleplex assays would guarantee maximum sensitivity, while multiplex assays would rather provide a broad panel of related, cross-reactive molecules for further definition of the IgE repertoire (9). Nevertheless, its use is not based on the clinical manifestations of the subjects and results are not a decisive predictor of clinical reactivity. Provocation tests help to confirm allergy to a suspected allergen, particularly when other tests cannot differentiate between clinically relevant sensitisation and allergic sensitisation not leading to relevant symptoms (10). Thus, it is important to keep in mind that sIgE tests always must be interpreted in the context of the patient´s history and outcome of challenge tests if needed (10).

**Figure 1:**
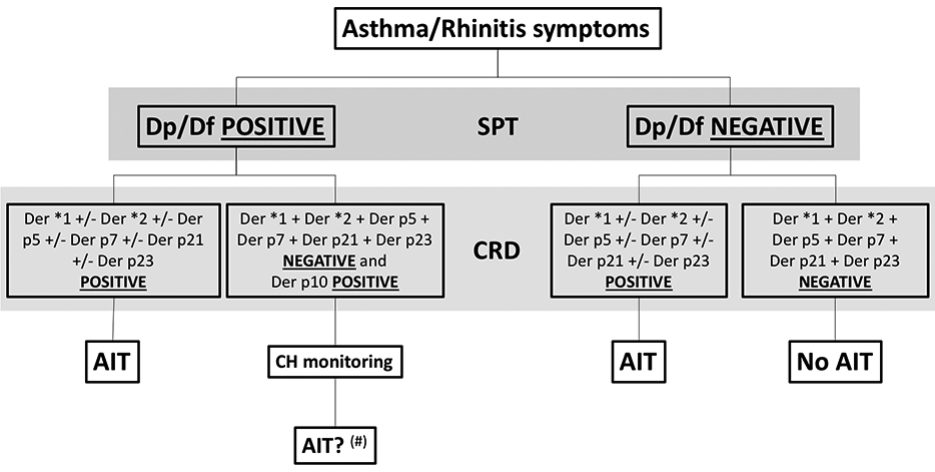
Suggested decision algorithm for mite allergic rhinitis/asthma in temperate countries. SPT, Skin Prick test; CRD, component resolved diagnosis; Dp, D. pteronyssinus extract; Df, D. farinae extract; AIT, allergen immunotherapy; CH, clinical history; +/−, and/or; +, and; Der*, Der p/Der f; (#), assuming the presence of well-characterized Der p10 in the extract.

A possible decision algorithm for mite allergy in our setting is the so-called top-down procedure, according to González-Pérez et al. (10, 11) (Figure 1). This method places the patient's clinical history first, on the basis of which, and after the use of a skin test with allergenic extracts of *Dermatophagoides pteronyssinus* and *Dermatophagoides farinae* and/or the corresponding determination of specific IgE against these sources, would allow us to move on to the next line of action, provided that the result obtained was positive (12–14). Positivity to the following molecular profile, Der p 1/Der p 2 and/or Der p 23, and/or Der p 5, and/or Der p 7 and/or Der p 21 allergens (10, 15–23) would lead to a recommendation for allergen-specific treatment and avoidance of the source (11).

Regarding the molecular components, described at least 10 years ago (17–23), of the proposed profile (11) and now available for routine diagnostics (24), some authors such as Walsemann et al. (16) and Yuriev et al. (15) demonstrated the clinical importance of sensitisation counts for certain allergens (Der p 5, Der p 20 and Der p 21), recalling that their incorporation into diagnostic measures would improve the diagnosis and risk assessment of HDM-allergic patients. A recent study developed in Tenerife (Spain) (8), found that most of the subjects (83.34%) with allergic rhinitis displayed a specific IgE response to more than eight mite HDM molecules, in contrast to patients with asthma (66.66%) or atopic dermatitis (66.66%), who showed a polysensitization profile to eight or more individual HDM allergens. In addition, the authors found specific IgE-binding responses to not only Der p 1, Der p 2, and/or Der p 23, but an increased sensitization rate to Der p 7 (50%) for patients with atopic dermatitis and asthma.

Certain situations require a complete molecular diagnostic work-up after analysing the clinical history and sensitisation test results. The U-shape approach, a top-down approach combined with a diagnostic from molecules to clinical implications (bottom-up), explores the degree and potential clinical relevance of further cross-reactivities to related molecules of a protein family (9).

## The allergenic source

Manufacturing diagnostic tests and anti-allergenic vaccines must be performed under optimal conditions. Thus, the allergen extracts used require to be of proven quality (25), so that after applying the corresponding methods and acceptance criteria that assess and guarantee the aforementioned quality, the clinician can work with the necessary sensitivity and specificity.

To this effect, the presence of the most relevant allergens of *Dermatophagoides pteronyssinus* and *Dermatophagoides farinae* (Der p 1/Der f 1, Der p 2/Der f 2, Der p 5/Der f 5, Der p 7/Der f 7, Der p 10/Der f 10, Der p 11/Der f 11, Der p 20/Der f 20, Der p 21/Der f 21 and Der p 23/Der f 23) should be demonstrated by mass spectrometry in the allergenic extracts, and group 1, 2 and 23 allergens should be quantified by double sandwich ELISA, using specific monoclonal antibodies.

## The patient

Precision medicine places the patient at the centre of the specialist's decision-making process, based on a plan adapted to the patient. Knowledge of the clinical and immunological phenotype of the population allows to establish criteria leading to a more rigorous diagnostic interpretation, so the objectives of AIT can be met.

In the 1990s, Sporik et al. (26) investigated the relationship between exposure to house dust mite allergen (Der p 1) and the development of sensitisation and asthma in a cohort of British children at risk of allergic disease due to family history, concluding that, in addition to genetic factors, early childhood exposure to house dust mite allergens was an important determinant of the subsequent development of asthma.

In 2022, Romero Sánchez et al. (27) found that Der p 23 was the predominant allergen in their study population (83.7%) despite the sIgE levels to Der p 1 and Der p 2 were higher than sIgE Der p 23 levels, and that the 8.2% of the patients were monosensitised to Der p 23. They suggested that Der p 23 may be a prevalent allergen in regions with high rates of HDM exposure and that its presence could increase the risk of asthma, even though Der p 23 sIgE levels to this allergen are generally low.

Gonzalez-Perez et al. (28) showed a clinically relevant sensitisation to HDM with moderate to severe persistent asthma, in which the major allergens Der p 1, Der p 2 and Der p 23 were recognized in >70% of all subjects, while Der p 5, Der p 7 and Der p 21 reached up to 51% of the cohort. In addition, a complex pleomorphic repertoire of IgE-recognised HDM molecules was observed, including 38 distinct profiles.

Muddaluru et al. (29), analysed the serum samples from 685 HDM-allergic subjects with allergic rhinitis and asthma from Canada, Europe, South Africa, and the USA, to determine the specific IgE levels of 17 HDM allergens. They found that sensitisation profiles differed considerably between geographical areas. The main sensitisers in all areas were the group 1 and 2 allergens, and Der p 23 (64% of the subjects had specific IgE to Der p 23, 2.3% of these were monosensitisations); In Africa, Der p 23 was the main sensitiser allergen (86% prevalence) and Der p 7 also showed to be a major allergen, with a 56% of sensitization.

This heterogeneity should be considered in the diagnosis and treatment of HDM-allergic patients.To this end, several studies have been proposed to understand the molecular exposome of the different regions, and to evaluate how the molecules of an allergenic source may influence the allergic pathology of the patients under study (15, 28, 30, 31). Gonzalez-Pérez et al. (32) described on a regional scale different patterns of molecular reactivity dependent on climate and other factors, in which the allergens Der p 21, Der p 5 and Der p 7 also appeared as serodominant molecules, in addition to the highly prevalent IgE responses to major HDM allergens, especially in subjects with asthma and atopic dermatitis.

An accurate characterisation of the external exposome at a molecular level and their putative role as clinically relevant allergens is essential to elucidate the phenotypic diversity of atopic disease, with a view to personalising diagnosis and therapy.

## Treatment

A “tailor-made” AIT using only genuine sensitisers seems difficult to realise, as there are too many different sensitisation profiles and so far, commercial extracts have shown to be effective, as well as purified/recombinant molecules for AIT. Allergen-specific AIT is a well-documented effective treatment for respiratory and Hymenoptera venom allergy (33). Although AIT was used empirically in the early 1900s, its efficacy has subsequently been corroborated by several controlled studies for both, the subcutaneous SCIT and sublingual SLIT routes of administration.

AIT has recently been described as a prototype of precision medicine (2), provided that some rules are respected: a correct diagnosis of the specific allergy, with standardised extracts for skin testing, and an available specific AIT product documented in controlled studies.

Since its appearance in the therapeutic armamentarium of allergic diseases more than 110 years ago, SIT has undergone numerous improvements related to allergen standardization, administration schedules, novel adjuvants, use of recombinant allergens (including hypoallergenic variants), etc. Likewise, in recent decades, effective and safe alternative routes of administration have emerged, such as the sublingual (SLIT) route. All this allows treatment to be personalized according to the patient's characteristics, needs and preferences. In any case, several key aspects should be considered when prescribing an SIT: efficacy, safety, cost, and convenience (34, 35). The impact of the treatment should be evaluated, not only on clinical and biological variables, but also on aspects related to quality of life. In regards of safety, SCIT has been classically associated with a greater risk of systemic reactions, while SLIT induces relatively frequent local reactions at the oropharyngeal level (36). In any case, modified extracts have very remarkable safety profiles that have drastically reduced the risk of potentially serious adverse reactions. Other aspect to consider is the cost-effectiveness of SIT, which has shown a clear long-term advantage compared to pharmacotherapy because, unlike the latter, subcutaneous and sublingual SIT has been shown to be able to modify the underlying cause of the disease, with proven long-term clinical benefits after cessation of treatment (36). Finally, treatment adherence is a crucial element in obtaining the desired effect in any form of treatment. In this regard, patient preferences must be considered to ensure that treatment compliance is optimal and, consequently, so are the results.

Because of its disease-modifying potential, AIT would be considered as the cornerstone of the cure for allergic diseases. Allergen immunotherapy faces challenges related to efficacy, safety, duration, and patient compliance, hence the demand for new vaccines to overcome these drawbacks. Some of these, currently rely on novel modalities such as mAb-based therapies, or new adjuvants that specifically target key nodes in the allergic inflammatory network (37).

**Figure 2:**
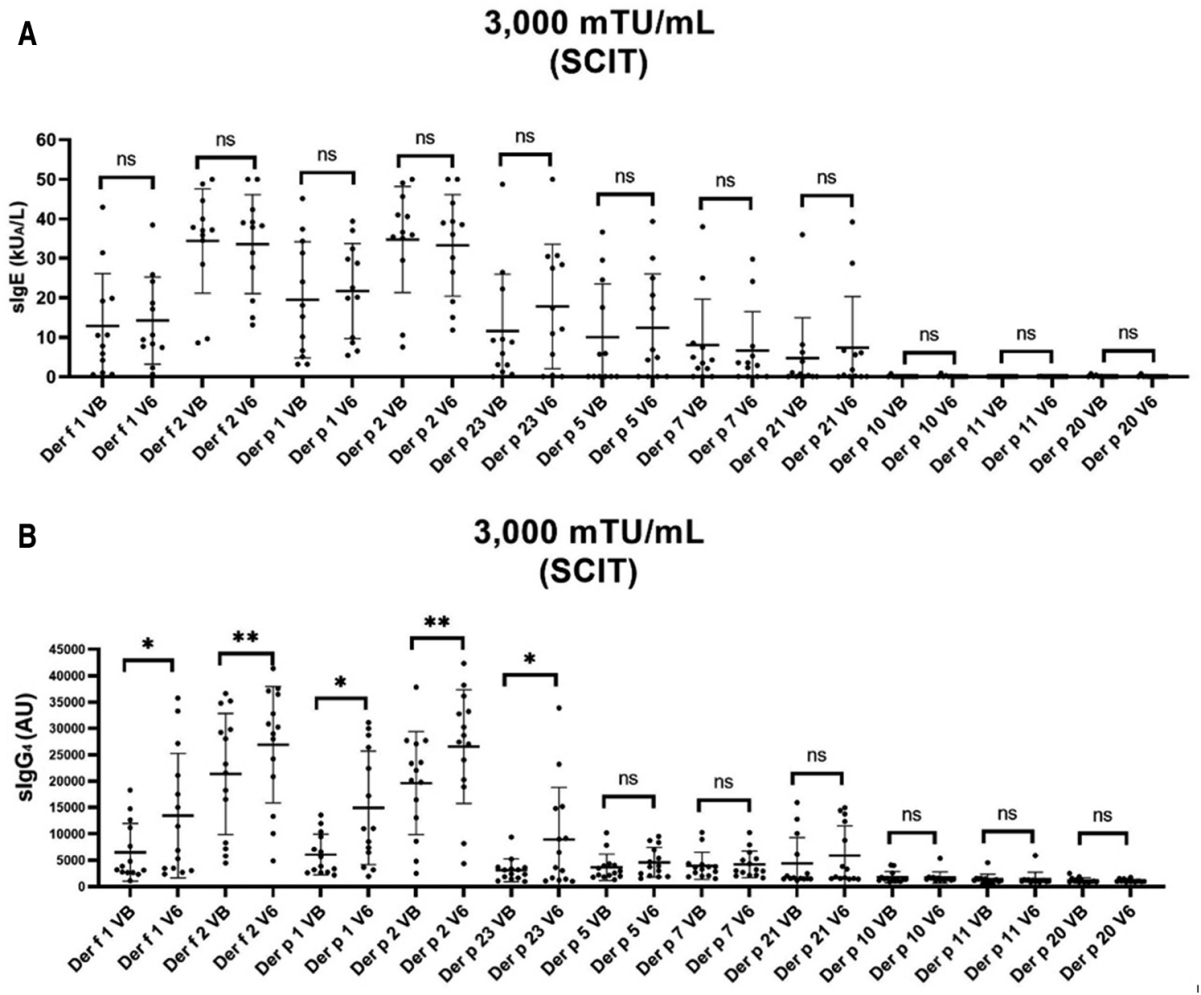
Molecular profile of sIgE and sIgG_4_ levels of mite allergic patients, before and after treatment with *D. pteronyssinus* and *D. farinae* polymerised extracts conjugated with mannan SCIT.

New AIT vaccines targeting dendritic cells generated by conjugation of allergoids with non-oxidised mannan have been developed (38–40). In the case of mite allergy treatment, a study has been carried out to find the optimal dose for both, subcutaneous (SCIT) and sublingual (SLIT) administration of polymerised extracts of *D. pteronyssinus* and *D. farinae*, conjugated with mannan in a double-blind, placebo-controlled study in patients with perennial allergic rhinitis. The results of the study showed that after 4 months, 62% and 61% of patients assigned to the 3,000 mTU/ml subcutaneous and sublingual group, respectively, improved after experiencing a positive nasal challenge at or above 3 times the initial concentration. Symptom/medication scores were reduced compared to placebo by 70% in subjects receiving SCIT and 40% in those receiving SLIT (41). Comparison of sIgE and sIgG_4_ levels of mite allergic patients, before and 6 months after treatment with SCIT *D. pteronyssinus* and *D. farinae* polymerised extracts conjugated with mannan, are shown in Figure 2.

The management of allergic disease should merge the different aspects encompassing the diagnosis, allergenic source, the patient, and treatment. Advances on molecular biology have led to the identification of major, mid-tier and minor allergens in the mite allergenic sources, which should be present in the allergenic extracts used in diagnosis and IT. The identification of these allergens has made it possible to identify the sensitisation profile of mite allergic patients, and some studies are beginning to deepen our understanding of allergen exposure (molecular exposome) and the relationship with clinical manifestations. Deep knowledge of all these aspects and their interrelation leads to a better approach to the mite allergic patient and thus, it helps to prescribe the best personalised treatment.

## Data Availability

The datasets presented in this article are not readily available because these are raw datasets that are pending publication in a more extensive form elsewhere. Requests to access the datasets should be directed to AN-G, antonio.nieto@me.com.
